# Early metabolic and transcriptional variations in fruit of natural white-fruited *Fragaria vesca* genotypes

**DOI:** 10.1038/srep45113

**Published:** 2017-03-22

**Authors:** Katja Härtl, Alisandra Denton, Katrin Franz-Oberdorf, Thomas Hoffmann, Melanie Spornraft, Björn Usadel, Wilfried Schwab

**Affiliations:** 1 Biotechnology of Natural Products, Technische Universität München, Liesel-Beckmann-Str. 1, 85354 Freising, Germany; 2 Institute for Biology I, RWTH Aachen University, Worringer Weg 3, 52074 Aachen, Germany; 3 Physiology Weihenstephan, TU München, Weihenstephaner Berg 3, 85354 Freising, Germany

## Abstract

Strawberry fruits (*Fragaria vesca*) are valued for their sweet fruity flavor, juicy texture, and characteristic red color caused by anthocyanin pigments. To gain a deeper insight into the regulation of anthocyanin biosynthesis, we performed comparative metabolite profiling and transcriptome analyses of one red-fruited and two natural white-fruited strawberry varieties in two tissues and three ripening stages. Developing fruit of the three genotypes showed a distinctive pattern of polyphenol accumulation already in green receptacle and achenes. Global analysis of the transcriptomes revealed that the ripening process in the white-fruited varieties is already affected at an early developmental stage. Key polyphenol genes showed considerably lower transcript levels in the receptacle and achenes of both white genotypes, compared to the red genotype. The expression of the *anthocyanidin glucosyltransferase* gene and a glutathione S-transferase, putatively involved in the vacuolar transport of the anthocyanins, seemed to be critical for anthocyanin formation. A bHLH transcription factor is among the differentially expressed genes as well. Furthermore, genes associated with flavor formation and fruit softening appear to be coordinately regulated and seem to interact with the polyphenol biosynthesis pathway. This study provides new information about polyphenol biosynthesis regulators in strawberry, and reveals genes unknown to affect anthocyanin formation.

Strawberry (*Fragaria* × *ananassa*) is one of the world’s most popular fruit crops with an annual production of more than 8.1 million tons in 2014 (http://faostat3.fao.org/browse/Q/QC/E). The strawberry is considered as “accessory fruit” because the fruit is not formed by the enlargement of the ovary but the edible, fruity pulp, also referred to as receptacle, is derived from adjacent tissue exterior to the carpel. The achenes (seeds) are distributed spirally across the epidermis of the pulp^1^. The development of the strawberry fruit is regarded independent of increased ethylene biosynthesis and respiration, which is why strawberries are considered non-climacteric fruits^2^, although recent studies suggest ethylene plays a role in strawberry fruit ripening^3^. The ripening process can be traced by observing the changes in fruit size and color. The stages are usually classified as green, white, turning, and red, and their development is accompanied by changing compositions of plant hormones and metabolites^4^. Strawberry fruits ripen quite fast within about 30 days. The plants are also small in size and easy to propagate. These qualities, together with an unusual fruit structure and color formation, have made the strawberry plant an advantageous model system to study fruit development. Woodland strawberry *F. vesca* has a small, sequenced genome (240 Mb), and is commonly used as a genetic model plant for the *Rosaceae* family and, in particular, the *Fragaria* genus.

Besides the appealing flavor, much of the attractiveness of strawberries is based on the bright red fruit-color caused by anthocyanin pigments^4^,^5^. Pelargonidin 3-O-glucoside, its 6′-O malonated derivative and cyanidin 3-O-glucoside are the major anthocyanins of strawberry fruit and are biosynthesized from phenylalanine by the phenylpropanoid-flavonoid-anthocyanin pathway, which has been thoroughly investigated by genetic, biochemical and metabolite profiling studies^6^. Anthocyanins are also associated with a large number of health-promoting effects. They possess anti-oxidative properties, have positive impacts on cardiovascular disorders and degenerative diseases, and are able to protect DNA integrity^7^,^8^,^9^. The basic biosynthetic pathway of anthocyanins is known, and most plant species share a large number of enzymatic reactions, although there are differences regarding the types of anthocyanins that accumulate^10^,^11^,^12^.

In contrast to the red-fruited *F. vesca* genotypes, there are also varieties that produce white fruits, even when they are fully ripened. This is not the result of continuous breeding or genetic modification, but a naturally occurring phenomenon. In the white-fruited varieties the pigment formation seems to be impaired by down-regulation of a single or multiple biosynthetic genes, or because an essential gene is non-functional. Key factors in the regulation of the flavonoid and anthocyanin pathway are MWB (MYB-bHLH-WD40) complex proteins^13^. In order to gain a deeper insight into the regulation of anthocyanin biosynthesis, we performed comparative metabolite profiling and transcriptome analysis of one red-fruited (*F. vesca* cv. Reine des Vallees (RdV)), and two white-fruited (*F. vesca* cv. Yellow Wonder (YW) and Hawaii 4 (HW4)) woodland strawberry varieties (Fig. 1) by liquid-chromatography coupled with mass spectrometry analysis (LC-MS), and RNA-sequencing (RNA-seq), respectively. To survey gene expression during fruit development we performed RNA-Seq on green, intermediate and ripe (white-ripe and red-ripe, respectively) fruits, and separated the achenes (seeds) from the receptacle (pulp). To determine the metabolic differences between the three genotypes, the level of anthocyanins and relevant precursors were analyzed by LC-MS, and the expression pattern of candidate genes was validated by qPCR.

Our analysis completes a recently published *F. vesca* transcriptome data set, which provides gene expression data of the fruit development stages from fertilized flower to big green fruit^14^,^15^. Thus, the transcriptome of the complete strawberry fruit ripening process from flower to ripe fruit is now available, with our data covering green to ripe developmental stages (Supplementary data File S1). The analyses of the white-fruited genotypes show that the phenylpropanoid/flavonoid/anthocyanin metabolism and the gene transcript levels are already perturbed at early developmental stages in YW and HW4.

## Results

### Metabolite Analysis

**Figure 1:**
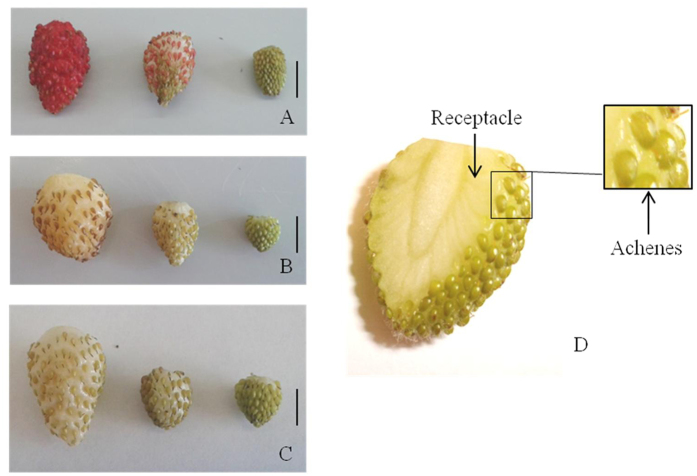
Fruits of *F. vesca* variety Reine des Vallees (**A**), Yellow Wonder (**B**) and Hawaii 4 (**C**) of the ripening stages ripe (left), intermediate (middle) and green (right). Cross-section of a green fruit of *F. vesca* Hawaii 4 (**D**), with arrows indicating the tissues (receptacle, achenes) separated before RNA and metabolite extraction. Scale bars = 5 mm.

**Figure 2:**
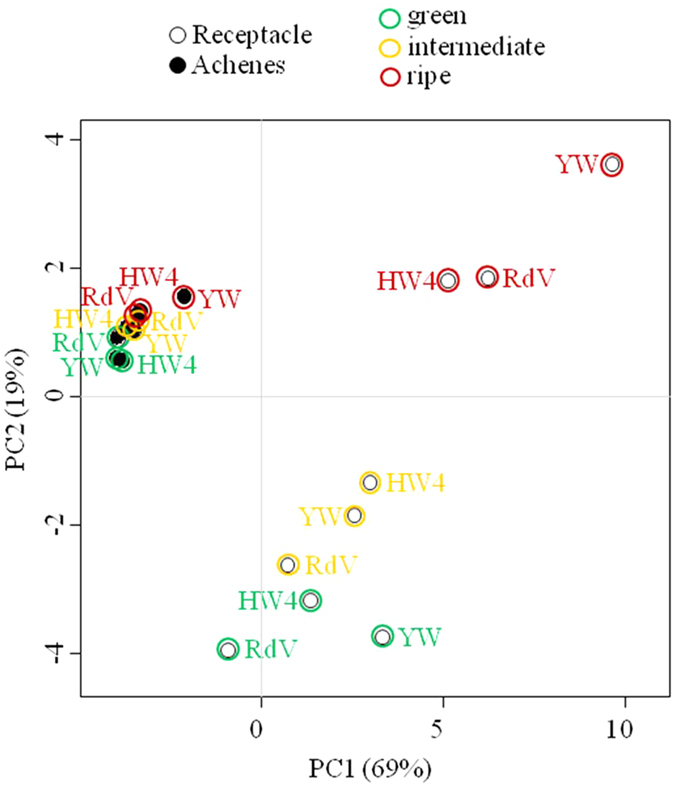
Untargeted analysis of secondary metabolites in receptacle and achenes of strawberry varieties RdV, YW, and HW4 of three developmental stages (green, intermediate, and ripe). 3–5 biological replicates per data point. The variance in the data is depicted by principal component analysis (PCA).

**Figure 3:**
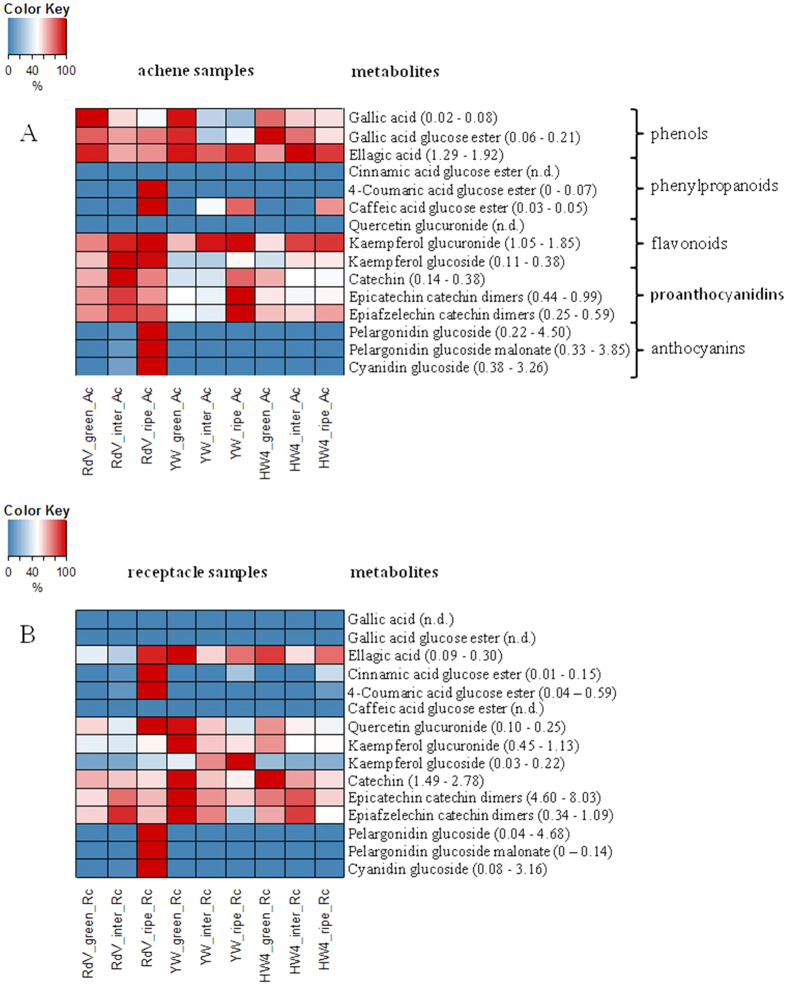
Quantification of secondary metabolites by LC-MS in achenes and receptacle of strawberry fruits. Heatmaps show levels that are expressed as per mil equivalents of the dry weight (relative concentration), with lowest levels shown in blue, and highest levels in red. Individual min. and max. values are given in parentheses. (**A**) Colour code presentation of metabolite levels in achene (Ac) tissues of different ripening stages (green, intermediate, ripe), and *F. vesca* varieties (RdV, YW, and HW4). (**B**) Metabolite levels in receptacle (Rc) tissues. n.d. not detected. Three to five replicates were analyzed.

Untargeted and targeted metabolite analyses of phenols, phenylpropanoids, flavonoids, proanthocyanidins, and anthocyanins were performed by LC-MS in receptacles and achenes of green, intermediate and ripe fruits of *F. vesca* RdV, YW and HW4 (Figs 2 and 3). 271 untargeted metabolites showed variance between the three genotypes (p-value ≤ 0.01). Their analysis uncovered much lower variance in the data sets of the metabolites found in the achenes of RdV, YW, and HW4 than in the data of the receptacles (Fig. 2). The receptacles of the three *F. vesca* varieties clustered according to their developmental stages. Thus, metabolites that confer the red color to ripe RdV fruits did not strongly contribute to the variance in the data. Overall, according to the untargeted analysis, the major metabolites were similar in the three genotypes at the identical ripening stage. However, the targeted metabolite analysis showed, in line with the color change of the receptacle and achenes of RdV (Fig. 1), high levels of anthocyanins such as pelargonidin glucoside, pelargonidin glucoside malonate, and cyanidin glucoside in ripe receptacle and achenes of RdV, but not in fruits of YW and HW4 (Fig. 3). Each developmental stage and fruit tissue is dominated by a certain group of phenolic compounds. For instance, phenols such as gallic acid, gallic acid glucose ester and ellagic acid were major metabolites in green achenes of RdV, whereas flavonoids were abundant in intermediate achenes of RdV, and anthocyanins and phenylpropanoids dominated in the late developmental stage. In most cases, the levels of secondary metabolites in achenes and receptacle of the white-fruited genotypes differed considerably from the concentrations determined in the respective tissues of RdV. Although, gallic and ellagic acid accumulated in green achenes of YW to levels found in green achenes of RdV, the concentrations of flavonoids were significantly reduced. In contrast, in green receptacles of YW, the levels of flavonoids even exceeded the concentrations detected in the same tissue of RdV. Thus, the differential metabolite levels suggest that changes in secondary metabolism reflect organ and developmental specificities.

### Global Analysis of the Fruit Transcriptome

Global mRNA sequencing of the receptacle and achenes of red-fruited *F. vesca* RdV variety, and both white-fruited *F. vesca* YW and HW4 varieties was performed to investigate the differential accumulation of transcripts. RNA was pooled from receptacles and achenes of ten fruits per sample to ensure high reliability regarding the stage of fruit ripening (Supplementary Table S1). Sequencing yielded 249,582,360 reads of 100 bp in length (Supplementary Table S2), giving ~25 billion nucleotides of total sequence data. After quality clipping, 245,603,827 reads were selected. The mapping of the selected reads to the *F. vesca* reference genome resulted in a total pool of 204,888,523 transcript counts (greater than 83% overall mapping rate, Supplementary Table S3). Subsequently, the read counts were normalized by DESeq2 size factors, and scaled to per million range (rpm: r*eads per million*). Genes with fewer than 20 normalized counts summed across samples were considered as not expressed. Out of 33673 annotated *F. vesca* genes, 19208 were expressed above this threshold.

**Figure 4:**
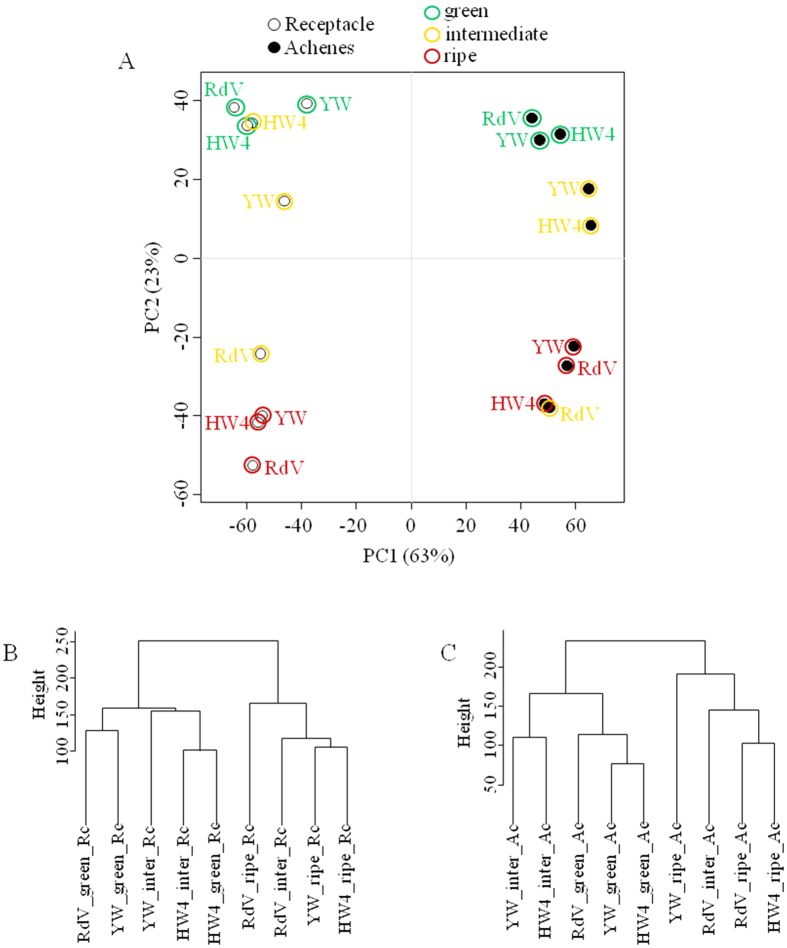
Global analysis of gene expression among samples and strawberry varieties. (**A**) Principle component analysis (PCA) of transcripts (top 500) in *F. vesca* varieties RdV, YW, and HW4, in two different tissues (achenes and receptacle) and of three ripening stages green, intermediate and ripe. (**B**) Cluster dendrogram showing global relationship among achenes samples. (**C**) Cluster dendrogram showing global relationship among receptacle samples.

To investigate global gene expression relationships, we performed a principle component analysis (PCA), and visualized the correlations also by dendrograms of the achenes and receptacle data sets (Fig. 4). When the 500 most highly expressed transcripts were employed for PCA analysis, the receptacles and achenes were clearly set apart (left and right), as well as the green and ripe developmental stages (top and bottom, Fig. 4A). The receptacles and achenes of the intermediate ripening stage of YW and HW4 grouped with the green tissues, whereas the receptacle and achenes of intermediately ripened RdV clustered with the ripe tissues of all varieties. Thus, gene expression of the white genotypes at the intermediate stage is more closely related to green unripe tissue, and the intermediate stage of the red genotype RdV to the ripe tissues. This observation was also confirmed by hierarchical clustering of the achenes and receptacle data (Fig. 4B and C).

### Analysis of differentially expressed genes between RdV and both white genotypes YW and HW4

To find candidate genes that might explain the loss-of-color phenotype in YW and HW4, differential expression between genotypes was assessed. Thirty-three genes were significantly down-regulated in white genotypes (YW, HW4) compared to the red genotype RdV (Table 1). Five genes encode enzymes with confirmed biochemical functions in *F. vesca* or *F.* × *ananassa*. Transcript levels of early (chalcone synthase *FaCHS2–2*: gene26826, chalcone isomerase *FaCHI*: gene 21346, and flavanone 3-hydroxylase *FHT*: gene14611), and late (dihydroflavonol reductase *DFR*: gene15176, anthocyanin synthase *ANS*: gene32347, and anthocyanin 3-*O*-glucosyltransferase *FaGT1*: gene12591) anthocyanin biosynthesis genes were equally reduced. Furthermore, gene31672 a predicted glutathione S-transferase is among the genes showing highest differential expression (logFC = −7.2), with transcripts accumulating almost exclusively in ripe and intermediate tissues of the red genotype RdV (Supplementary data File S1).

On the contrary, 31 genes were significantly up-regulated in both white genotypes (Table 2). Many candidates currently lack a functional prediction but show remarkable differences between the genotypes. For example candidate gene20847 (logFC = 10.7), almost not expressed in any tissues of RdV exhibits very high expression (up to 1,785 RPM) in tissues of HW4, and lower but considerable expression in tissues of YW. Furthermore, gene27422 a predicted transcription factor ORG2-like of the bHLH class is exclusively expressed in the white genotypes.

### Transcript level profiles of known anthocyanin and flavonoid pathway genes during strawberry fruit development

**Figure 5:**
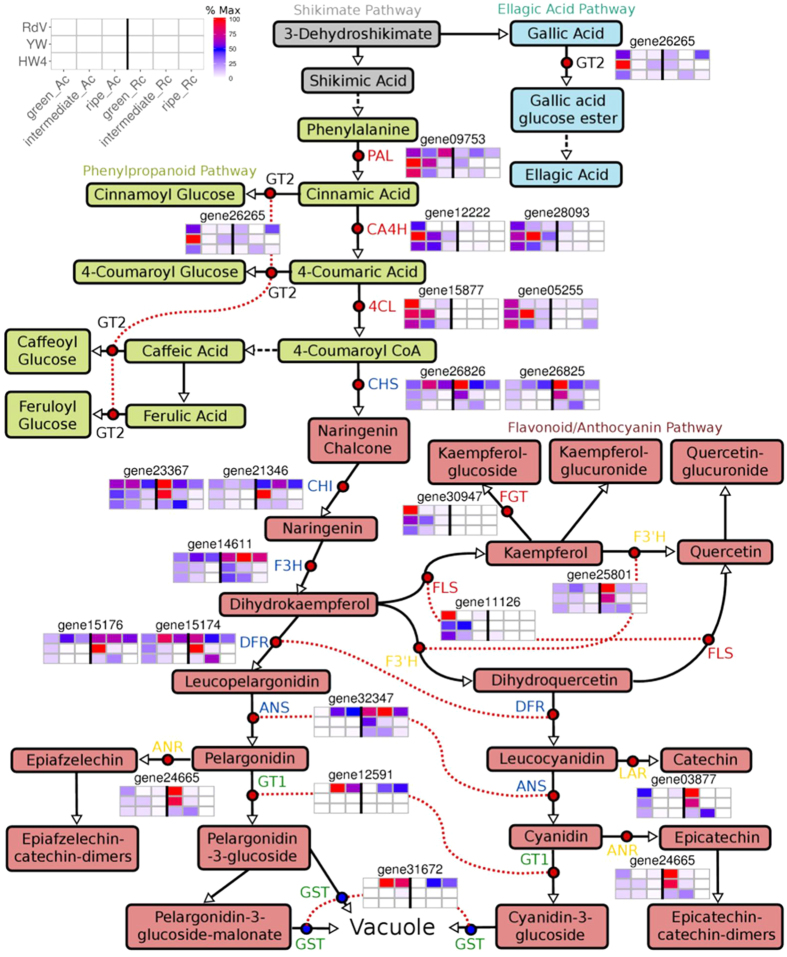
Schematic illustration of the shikimate, ellagic acid, phenylpropanoid, flavonoid and anthocyanin pathway. Red dots indicate biochemically characterized enzymes in strawberry fruit: ANS, anthocyanidin synthase; ANR, anthocyanidin reductase; CA4H, cinnamic acid 4-hydroxylase; CHI, chalcone isomerase; CHS, chalcone synthase; 4CL, 4-coumaroyl-CoA ligase; DFR, dihydroflavonol reductase; FGT, flavonoid glucosyltransferase; F3H, flavanone 3-hydroxylase; F3′H, flavonoid 3′-hydroxylase; FLS, flavonol synthase; GT1, anthocyanidin glucosyltransferase; GT2, (hydroxy)cinnamic acid and (hydroxy)benzoic acid glucosyltransferase; LAR, leucoanthocyanidin reductase; PAL, phenylalanine ammonia lyase. Blue dots indicate putative GST, glutathione S-transferase. Identical genes are connected by a dotted red line when adjacent. Enzymes shown in the same color are co-regulated. Heatmaps show relative transcript levels (% Max) of genes in receptacle (Rc) and achenes (Ac) of *F. vesca* RdV, YW, and HW4 at the green, intermediate and ripe developmental stage.

Next, we analyzed the transcript level profiles of anthocyanin and flavonoid pathway genes, whose encoded proteins have already been biochemically characterized. Gene expression levels in achenes and receptacle of RdV, YW, and HW4 of three developmental stages (green, intermediate and ripe) were compared (Fig. 5). Four groups of genes could be clearly distinguished by means of their transcript profiles. Early anthocyanin and flavonoid pathway genes such as *PAL, CA4H, 4CL, FLS*, and a flavonoid glucosyltransferase (FGT) gene show high expression levels in green achenes of RdV, YW, and HW4 as well as in achenes of YW and HW4 of the intermediate ripening stage (Fig. 5, Supplementary Figures S1 and S2). *CHS, CHI, F3H, DFR*, and *ANS* formed the second group. Transcript abundance of these genes was high in achenes and receptacles of RdV of all developmental stages but low in fruit of YW and HW4, except for green achenes and receptacle, and intermediate receptacle. Gene transcript levels of the first two groups differed considerably between the red- and white-fruited genotypes in achenes and receptacle at the intermediate (and ripe) developmental stage. However, the most significant difference was observed for the mRNA abundance of the anthocyanidin glucosyltransferase gene *FaGT1* and an uncharacterized glutathione S-transferase gene. Both were exclusively expressed in fruit of RdV at the intermediate and ripe developmental stage. *F3*′*H, ANR*, and *LAR* formed the fourth group. They were primarily expressed in the green fruit of the three genotypes, and in intermediate receptacle of HW4. The transcript profiles of *F3*′*H, ANR*, and *LAR* in fruit of HW4 was clearly different from that of RdV and YW. Thus, gene expression perturbation of flavonoid pathway genes in HW4 occurs already in the green developmental stage. The glucosyltransferase gene *FaGT2* was mainly expressed in green achenes of the three genotypes, and ripe receptacles of RdV.

### Expression levels of genes involved in fruit softening and flavor formation

**Figure 6:**
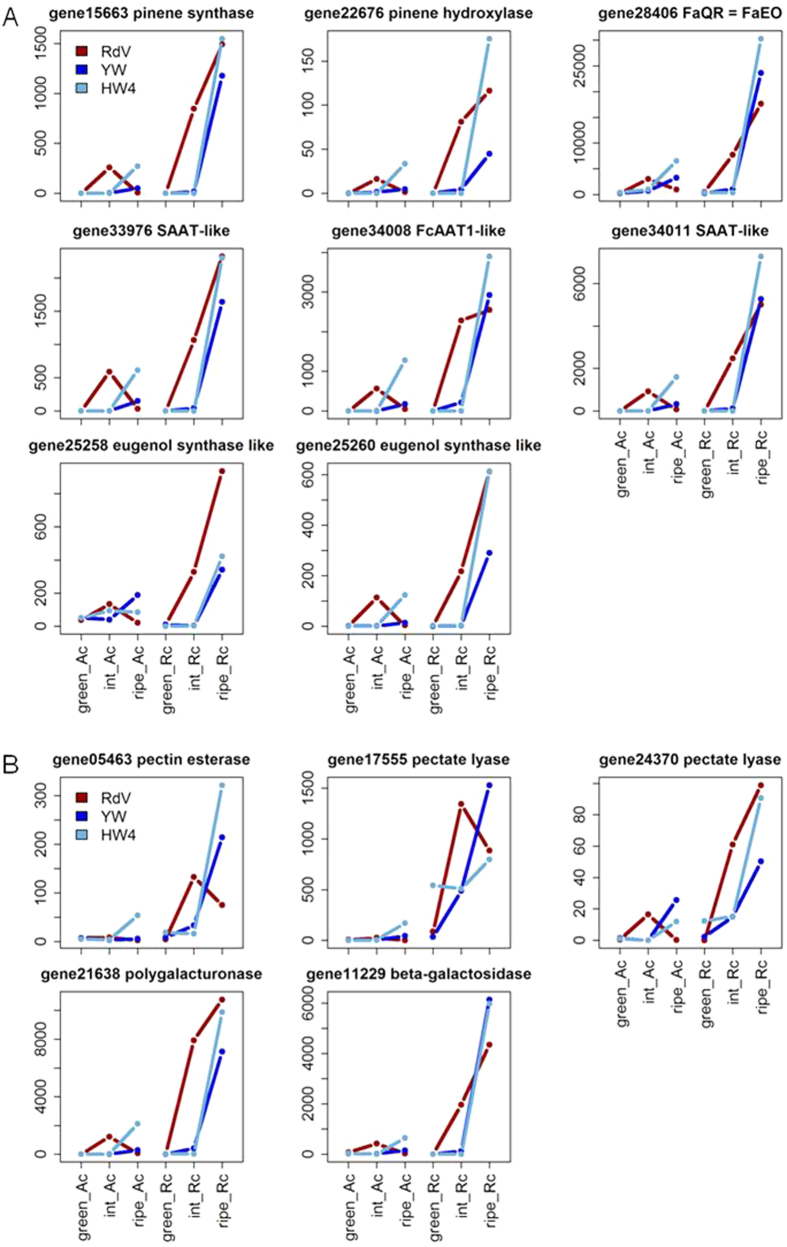
Transcript levels (normalized RPM) of genes encoding enzymes involved in fruit flavor formation (**A**) and softening (**B**) in receptacle (Rc) and achenes (Ac) of *F. vesca* RdV, YW, and HW4 at the green, intermediate (int) and ripe developmental stage.

Finally, we wanted to know whether the impaired anthocyanin pathway in YW and HW4 affects the expression of genes involved in fruit flavor formation, and fruit softening. Transcript levels of well-characterized genes associated with volatile terpene (pinene synthase and hydroxylase), ester (acyltransferases FcAAT1, and SAAT), furaneol (FaQR), and eugenol (eugenol synthase) formation, as well as genes affecting fruit softening (pectin esterase, pectate lyase, polygalacturonase and beta-galactosidase) were analyzed in the data sets of RdV, YW, and HW4 (Fig. 6). The genes showed a similar ripening-related expression profile in the receptacles of the three genotypes, peaking at the ripe stage. It seems that the ripening process is slowed down in the white-fruited genotypes in comparison to RdV, because transcripts of genes involved in fruit flavor production, and degradation of cell wall polysaccharides are already abundant in receptacle of RdV at the intermediate stage, whereas these genes are almost solely expressed in ripe receptacle of YW and HW4.

## Discussion

Considerable information on the polyphenolic composition of commercial strawberry fruit (*F.* × *ananassa*)^16^,^17^,^18^ and woodland strawberry *F. vesca* fruit^19^,^20^,^21^ exist. However, data on the levels of phenolics in developing *F. vesca* fruits is missing, completely. The bright color of red-fruited strawberries is due to four major anthocyanins, pelargonidin 3-glucoside, pelargonidin 3-glucoside 6′-malonate, pelargonidin 3-rutinoside and cyanidin 3-glucoside^22^,^23^,^24^,^25^, which are formed by the phenylpropanoid/flavonoid/anthocyanin pathway during fruit ripening^6^,^26^,^27^. In white colored strawberries, these anthocyanins are reduced in the receptacle, and in some cases also in the achenes^28^,^29^,^30^. Similarly, only trace amounts of pelargonidin 3-glucoside, pelargonidin 3-glucoside 6′-malonate, and cyanidin 3-glucoside were detected in the ripe receptacle and achenes of YW and HW4; in contrast to their high abundance in fruit of RdV (Fig. 3). Untargeted analysis of secondary metabolites by PCA separated the achenes from the receptacles, whereas the receptacles were further subdivided according to their ripening stage (Fig. 2). Ripe fruit of RdV, YW, and HW4 clustered in the PCA plot, but can be readily differentiated by the different pigmentation (Fig. 1). Thus, the anthocyanin level in RdV fruit is not the primary variance in the data.

Although, green achenes of RdV, YW, and HW4 accumulated comparable levels of gallic acid, gallic acid glucose ester, and ellagic acid, the immature seeds of the three genotypes can be clearly differentiated by their varying flavonoid concentration (Fig. 3). Achenes of RdV exhibited a tri-phasic polyphenol accumulation profile. The levels of phenols, flavonoids, and anthocyanins/phenylpropanoids peaked in green, intermediate, and ripe seeds of the red-fruited genotype, respectively. During ripening of YW and HW4, flavonoids did not reach the concentrations found in RdV, except for kaempferol glucuronide in intermediate and ripe achenes of both white-fruited genotypes, and epicatechin catechin and epiafzelechin catechin dimers in ripe achenes of YW. In addition, the total amount of polyphenols is reduced in the white-fruited genotypes. Receptacles of RdV showed a bi-phasic formation of polyphenols, as flavonoid levels peak at the intermediate ripening stage, and anthocyanins, quercetin glucuronide, phenylpropanoids, and ellagic acid are abundant in the ripe pulp. In contrast, receptacles of YW contained high levels of ellagic acid and flavonoids with declining concentrations during ripening, except kaempferol glucoside. HW4 displayed a mixed pattern of polyphenol accumulation in the pulp, whereas the lowest levels were found at the ripe developmental stage. Overall, the divergent profiles of secondary metabolites suggest an interference of the pathway in the white-fruited genotypes YW and HW4 at an early developmental stage.

In addition to metabolite profiling, gene transcript abundance was quantified by RNA-seq analysis in achenes and receptacles of the red- and white fruited varieties during fruit ripening. Analysis of global gene expression by PCA separated the achenes from the receptacles, as well as green from ripe tissues (Fig. 4A). Intermediate receptacle and achenes of YW and HW4 clustered with green fruit samples, and intermediate pulp and seeds of RdV with ripe fruit samples. This indicates that variance in gene expression is highest between samples of the intermediate ripening stage, and confirms the hypothesis that the ripening process in YW and HW4 is already affected at an early stage. In contrast, the untargeted analysis of the metabolites did not show equal variance, as the achene samples of all genotypes grouped together (Fig. 2). On the other hand, ripe receptacle of RdV, YW and HW4 grouped together in the PCA of the transcripts (Fig. 4A), similar to the PCA of secondary metabolites (Fig. 2).

Analysis of differentially expressed genes revealed that expression of major genes in the anthocyanidin/flavonoid biosynthesis pathway was down-regulated in the white varieties (Table 1). The expression of the branch point gene *CHS* (gene26826 polyketide synthase 1) was severely reduced. *CHS* expression is known to be associated with fruit coloring, because artificial down-regulation of *CHS* function via antisense and RNAi methods leads to pigment loss in flowers or fruits of different plant species^31^,^32^,^33^,^34^,^35^. Furthermore, five genes acting downstream of *CHS* were also clearly down-regulated (Table 1). The protein encoded by CHI (gene21346 chalcone-flavonone isomerase 3) catalyzes the conversion of naringenin chalcone to the flavanone naringenin, thereby producing the basic skeleton of all flavonoid metabolites^10^. The protein encoded by *FHT* (gene14611 naringenin, 2-oxoglutarate 3-dioxygenase) oxidizes the central B ring of the flavanone naringenin to produce dihydrokaempferol^10^. The enzyme encoded by *DFR* (gene15176 bifunctional dihydroflavonol 4-reductase/flavanone 4-reductase) reduces dihydrokaempferol to colorless leucoanthocyanidins^36^,^37^. The polypeptide encoded by *ANS* (gene32347 leucoanthocyanidin dioxygenase) generates colored anthocyanidins like pelargonidin^38^, and the protein encoded by *FaGT1* (gene12591 anthocyanidin 3-O-glucosyltransferase 2) stabilizes the anthocyanidins by glucosylation^27^. The resulting anthocyanins accumulate, and are responsible for the coloring of fruits and flowers^39^. Thus, formation of anthocyanin precursors is considerably hampered in the white varieties. Among the significantly down-regulated candidates is also gene31672, a glutathione S-transferase (GST, Table 1) orthologous to GST Solyc02g081340 from tomato (*Solanum lycopersicum*). Transgenic tomato fruits exhibiting higher anthocyanin content showed increased expression of Solyc02g081340^40^,^41^, whereas expression in the *anthocyanin absent* mutant was barely detectable^42^. It has been proposed that anthocyanins might be transported into vacuoles via the noncovalent activity of GSTs^43^. Several *GST* genes with such functions have been characterized in plants including the *TT19* gene (encoding a type I *GST*) of Arabidopsis, *Bronze-2* (encoding a type III *GST*) of maize, *AN9* (encoding a type I *GST*) of petunia^44^,^45^,^46^ and two *GST* genes from grape^47^. Therefore, gene31672 might act in anthocyanin transport.

In addition to genes down-regulated in the white genotypes, also candidates significantly up-regulated were found (Table 2), such as a yet uncharacterized transcription factor (TF) of the bHLH class (gene27422 ORG2-like). It is widely acknowledged that TFs of the MYB and bHLH protein classes regulate the expression of anthocyanin biosynthesis genes^48^,^49^. MYBs linked to the anthocyanin pathway possess a highly conserved DNA-binding domain, which usually comprises two repeats (R2R3)^49^, and are suggested to interact with bHLH TFs^48^. Both, activators and repressors are known^50^,^51^. The bHLH proteins have not been extensively studied in plants. Those that have been characterized function in anthocyanin biosynthesis, phytochrome signaling, globulin expression, fruit dehiscence, and carpel and epidermal development^52^,^53^. Consequently, gene27422 could encode a bHLH TF regulating pigment formation in strawberry fruit. Recently, MYB10 (encoded by gene31413) was characterized as positive regulator of anthocyanin biosynthesis in *F.* × *ananassa*^50^, and *F. vesca*^54^. RNAi-mediated down-regulation of *MYB10* resulted in significant reduction of anthocyanin concentration in ripe receptacle of red-fruited *F.* × *ananassa*, and *F. vesca* varieties, while over-expression resulted in dark red fruits^50^,^54^,^55^. Furthermore, recent transcriptomic and SNP variant analysis revealed a single amino acid change in the MYB10 protein of the white-fruited varieties YW and HW4 that could be responsible for the loss-of-colour phenotypes^56^. Our data showed that *MYB10* transcripts were more abundant in ripe receptacles of the white-fruited varieties YW and HW4 than in red-fruited RdV (Supplemental Figure S3A) contradicting the observation that MYB10 is not differentially expressed in YW in comparison to the red-fruited *F. vesca* variety Ruegen^57^. Although MYB10 seems to be an important regulator of anthocyanin biosynthesis in receptacle, our data indicated that MYB10 might also have a significant role in ripe achenes due to the high transcript level. However, metabolic and transcriptional variations in fruit of natural white-fruited *Fragaria vesca* genotypes were already found at early developmental stages where *MYB10* is almost not transcribed. Therefore, additional transcriptions factors might account for these differences. MYB1, a transcriptional repressor in regulating the biosynthesis of anthocyanins in strawberry^29^,^51^,^58^, was not among the differentially expressed genes (Supplemental File S1). This indicates that MYB1 might not be the anthocyanin biosynthesis repressor responsible for the loss-of-color phenotype in the white-fruited *F. vesca* genotypes.

Amongst the differentially expressed genes (Tables 1 and 2) are candidates featuring comparable RPM levels in both white genotypes, such as gene01839 and gene13191. In contrast, other candidates showed diverging levels, such as gene30676 and gene30960. This indicates that in addition to metabolic differences among the two white genotypes (Fig. 3), also transcriptional differences can be found.

The general biosynthesis pathway of anthocyanins has been thoroughly investigated at both, the biochemical and the genetic level, in particular in *A. thaliana* and *Vitis* sp.^49^,^59^. Also in the *Fragaria* genus the key flavonoid pathway genes have been cloned, and their encoded proteins functionally characterized^6^. Enzymatic analysis of PAL, CHS/CHI, F3H, FLS, flavonoid 3-O-GT, and flavonoid 7-O-GT activity in crude fruit extracts demonstrated two distinct activity peaks during fruit ripening at early and late developmental stages for all enzymes except FLS^60^. The high activity at the immature stage corresponds to the formation of flavanols, while the second peak is clearly related to anthocyanin and flavonol accumulation^60^. According to our data, a biphasic transcript expression pattern for the flavonoid pathway genes was not observed (Fig. 5). Instead, genes could be grouped into classes according to their expression profiles in receptacle and achenes. Transcripts of key genes of the phenylpropanoid pathway (*PAL, CA4H*, and *4CL*) were highly abundant in immature seeds of *F. vesca*, and their expression profile suggests a coordinated transcriptional regulation in receptacles and achenes during fruit ripening (Fig. 5). At the gateway of primary metabolism *PAL, CA4H*, and *4CL* play a pivotal role as they are producing the precursor molecules of all polyphenols, including lignin. High degree of coordination in the overall expression of these three genes has been shown in parsley leaves, and cell cultures treated with UV light or fungal elicitor^61^. The gene expression profile of *FLS* and a putative flavonoid *GT* (gene30947 FGT) matched the transcript expression pattern of the phenylpropanoid genes, but act more downstream in the flavonoid pathway (Fig. 5). The fruit ripening program in red-fruited RdV is characterized by down-regulation of *PAL, CA4H*, and *4CL* in achenes of the intermediate developmental stage, which did not occur in seeds of YW and HW4. Thus, in immature fruit the early polyphenol biosynthesis pathway is already differently regulated in the red- and white-fruited genotypes. Similarly, flavonoid genes (*CHS, CHI, F3H, DFR*, and *ANS*) were coordinately expressed in a spatial and temporal manner (Fig. 5). They are involved in the supply of precursor molecules for proanthocyanidin, flavonoid, and anthocyanin production. In apple fruit, the anthocyanin biosynthetic genes, *CHS, F3H, DFR*, and *ANS*, are coordinately expressed during red coloration in skin, and their levels of expression are positively related to anthocyanin concentration^62^. Transcript levels of the flavonoid genes were particularly abundant in green receptacle of YW in comparison to HW4 (Fig. 5) and might, therefore, contribute to the high levels of flavonoids and proanthocyanidins in green pulp of YW (Fig. 3). The differential expression of the anthocyanidin glucosyltransferase gene *FaGT1*^27^ (gene12591), in the red-fruited and white-fruited *F. vesca* genotypes is striking (Fig. 5). Similarly, white-colored grape cultivars appear to be lacking anthocyanins because of the absence of an anthocyanidin *GT*^63^. In apple fruits, the transcript expression level of an anthocyanidin *GT* is positively related to anthocyanin concentration, and the gene is coordinately expressed with *CHS, F3H, DFR*, and *ANS* during red coloration in apple skin^62^. Moreover, late genes in the anthocyanin biosynthetic pathway are coordinately expressed during red coloration of litchi fruits, where low expression of *DFR* and *GT* result in absence or extremely low anthocyanin concentrations^64^. Interestingly, the transcript expression pattern of the putative *GST* candidate gene (gene31672) matched exactly the expression of *FaGT1* (Fig. 5), emphasizing its putative role in anthocyanin transport. The transcript expression profile of *FaGT2* (gene26265), a gene encoding a (hydroxyl)cinnamate GT^65^ suggests that GT2 might contribute to the production of gallic acid glucose ester (Fig. 3) in early developmental stages and to the production of (hydroxyl) cinnamic acid glucose esters in stages^66^.

The formation of red pigments would require the maintenance of high expression levels of *CHS, CHI, F3H, DFR*, and *ANS* in the receptacle and achenes, as well as the down-regulation of *PAL, CA4H*, and *4CL* in achenes of the intermediate ripening stage. In the white-fruited *F. vesca* genotypes, transcript expression profiles of the proanthocyanidin biosynthesis genes (*F3*′*H, ANR*, and *LAR*) and the pattern of flavonoid genes (*CHS, CHI, F3H, DFR*, and *ANS*) were coordinately regulated, and their ripening program appears to be unable to switch from the biosynthesis of flavonoids and proanthocyanidins occurring at the early stage to the production of anthocyanins in later stages. The polyphenol biosynthesis pathway in fruit of HW4 seems to be disturbed at an even earlier stage, as proanthocyanidin biosynthesis genes are already weakly expressed in green receptacle of HW4.

The interaction of polyphenol metabolism and fruit flavor formation has been frequently demonstrated as phenolic compounds can act as precursors of flavor molecules^67^. Thus, expression profiles of functionally characterized flavor biosynthesis genes pinene synthase and hydroxylase^68^, AAT^69^, QR^70^, and eugenol synthase^67^ were analyzed in the three genotypes (Fig. 6A). Transcripts of flavor genes were already abundant in red-fruited RdV at the intermediate fruit ripening stage, whereas in YW and HW4 mRNA of genes involved in flavor formation were only detectable at the ripe stage. It appears that the ripening program in the white-fruited genotypes is delayed, which is also supported by the comparison of the transcript profiles of the fruit softening genes pectin esterase^71^, pectate lyase^72^, polygalacturonase^73^, and beta-galactosidase^74^ in RdV and YW, as well as in HW4 (Fig. 6B). Overall, the flavor and softening genes seem to be coordinately regulated. It has been shown, that ripe fruits of red and white *F. vesca* varieties share most volatile organic compounds. Varying levels among the genotypes occur, but the main compounds such as esters presumably formed by AATs are present^75^.

## Material and Methods

### Plant Material

*F. vesca* Reine des Vallees (RdV), Yellow Wonder (YW) and Hawaii 4 (HW4) are three botanical forms of *F. vesca*, all of which produce small-sized plants and propagate without runners, except HW4. RdV has fruits with red flesh and red skin, whereas YW and HW4 fruits have both yellow flesh and skin (Fig. 1). The genetic and growth characteristics of YW and HW4 have been described^56^. *F. vesca* cv. RdV, YW and HW4 strawberry plants were grown at the Call Unit for plant research (Technische Universität München, Germany). Fruits were harvested in three ripening stages green [~10 days post-anthesis (DPA)], intermediate [~25 DPA], and ripe [~35 DPA] according to literature^4^,^26^. Fruits were sampled between May and August 2014/2015, frozen in liquid nitrogen directly after harvest, and stored at −80 °C until further usage (Fig. 1).

### Chemicals

Except where otherwise stated, chemicals were purchased from Sigma-Aldrich (Steinheim, Germany), Fluka (Steinheim, Germany) or Roth (Karlsruhe, Germany).

### RNA-Isolation, -Quantity and -Quality Assessment

For RNA isolation achenes were separated from the pulp, and each sample was ground to a fine powder by mortar and pestle. Three woodland strawberry varieties (*Fragaria vesca* cv. RdV, YW, and HW4), three fruit ripening stages (green, intermediate, ripe) and two tissues (achenes, receptacle), in total 18 different samples were processed. For each sample the RNA of 10–15 fruits was pooled. Total RNA was extracted according to the CTAB protocol (Liao *et al*., 2004). DNA was removed by treatment with RNase-free DNase I (Thermo Fisher Scientific Inc., Germany) for 1 h at 37 °C. RNA yields were measured and the RNA Integrity Number (RIN, Supplementary Table S1) was determined on a Bioanalyzer 2100 (Agilent Technologies, Germany) equipped with a RNA 6000 Nano Kit.

### RNA Sequencing and Library Preparation

Total RNA was sent to Eurofins Genomics (Germany), where RNA sequencing and library preparation was carried out. The 3′ fragment cDNA library was generated through fragmentation of total RNA by ultrasound before poly(A)-tailed 3′-RNA fragments were isolated using oligo-dT chromatography. Then, an RNA adapter was ligated to the 5′-ends of the poly(A)-tailed RNA fragments. First-strand cDNA synthesis was performed using an oligo(dT)-adapter primer and reverse transcriptase. The resulting cDNA was PCR-amplified using a high fidelity DNA polymerase. Each final cDNA library was purified, size selected, quantified and analyzed by capillary electrophoresis before RNA-Seq analysis was performed on an Illumina HiSeq2000 platform (Illumina Inc., USA). A PhiX library (Illumina Inc., USA) was added before sequencing to estimate the sequencing quality. Reads were processed by the CASAVA 1.8 package. Sequencing results are summarized in Supplementary Table S2.

### Quality Trimming, Mapping and Data Normalization

RNA-seq data processing was performed on Galaxy, a free public server that was installed locally^76^,^77^,^78^. The Application Programming Interface (API) and the Galaxy Data Manager were used for automation of the pipeline analyses^79^, and handling of built-in reference data^80^, respectively. The bioinformatics tools were installed and organized via the Galaxy ToolShed^81^. http://www.rosaceae.org/Reads were trimmed using the Trimmomatic tool^82^ with default settings for single end reads. The TruSeq3 adapters were removed in an initial ILLUMINACLIP step. Quality trimming was performed with a SLIDINGWINDOW step, and finally reads below 20 bp were discarded with a MINLEN step. Before and after trimming, the overall data quality was evaluated with the FastQC software (quality control tool for high throughput sequence data http://www.bioinformatics.babraham.ac.uk/projects/fastqc/).

Trimmed reads were aligned to the *F. vesca* reference genome (version 2.0.a1 downloaded from Genome Database for Rosaceae, GDR, www.rosaceae.org^83^) with TopHat^84^ using default settings. The results of the read mapping are summarized in Table S3. Aligned reads were quantified using HTSeq-count^85^ in “Union” mode for stranded reads with a minimum alignment quality of 10. The gene prediction input file was downloaded from GDR^83^. As poly A-tail selection was performed after fragmentation of the RNA, reads were derived from only the 3′ ends of transcripts and normalization by gene length to *Reads Per Kilobase of exon per Million mapped reads* (RPKM) or *Transcripts per Million* (TPM) would be inappropriate. Instead, the raw read counts were normalized for library size using the DESeq2 R package^86^, and adjusted to per million scale (divided by total normalized counts for all samples, times 18 for sample number, times 1,000,000), to produce normalized rpm.

### Differential Expression

Deferentially expressed genes were defined using the general linear models in edgeR^87^. Specifically, models were fitted with a factor for tissue, stage, and mature color of genotype and likelihood ratio test was performed comparing the white genotypes (YW and HW4) to RdV. The false discovery rate (FDR) was calculated according to^88^ and genes with FDR < 0.05 were considered significant. Accession numbers of flavonoid genes differentially expressed in the red- and white-fruited genotypes and transcription factors analyzed in this study are summarized in Table 3.

### Global Sequencing Data Analysis

The data were analyzed in R (R Core Team, 2015), employing appropriate packages mostly accessed via the open source software framework Bioconductor^89^. Before the cluster dendrogram was generated, the dataset was transformed using variance stabilization^90^. Subsequently, hierarchical clustering was performed using the complete method and Spearman distance metric. The PCA analysis was performed according to ref. 91. For assignment of functional gene predictions, MapMan “BINs”^92^ and open-source *F. vesca* gene ontology (GO) annotation^93^,^94^ were used.

### Metabolite Extraction

50 mg of lyophilized fruit powder was weighed (n = 3–5). The resulting samples were extracted with 250 μl of internal standard solution (0.2 mg ml^−1^ biochanin A and 4-methylumbelliferyl-*β*-D-glucuronide in methanol) and 250 μl methanol. After vortexing (1 min), sonication (10 min), and centrifugation (10 min, 16,000 g) the supernatant was collected. The residue was re-extracted with 500 μl methanol, and the supernatants were combined and dried in a vacuum concentrator. The secondary metabolites were re-dissolved in 50 μl of water, vortexed, sonicated and centrifuged. The clear supernatant was used for LC-MS analysis.

### Liquid chromatography-mass spectrometry (LC-MS)

Levels of secondary metabolites were determined on an Agilent 1100 HPLC/UV system (Agilent Technologies, Germany) equipped with a reverse-phase column (Luna 3 u C18(2) 100 A, 150 × 2 mm; Phenomenex, Germany), a quaternary pump, and a variable wavelength detector. Connected to the HPLC was a Bruker esquire3000plus ion-trap mass spectrometer (Bruker Daltonics, Germany). HPLC and mass spectrometry were performed at optimized conditions^33^,^95^. Resulting data were analyzed with Data Analysis 5.1 software (Bruker Daltonics, Germany), and metabolites were identified using the in-house database. Levels (per mil equivalents of the dry weight, ‰ equ. dw.) of secondary metabolites quantified during targeted analyses are summarized in Supplementary Tables S4 and S5.

### Untargeted Metabolite Data Analysis

Untargeted metabolite profiling analysis of the LC-MS data set was done according to published reports^96^,^97^,^98^ in R (Fig. 3). Peaks were grouped together across samples after correction of retention time deviations. After integration of the peak areas, the Wilcoxon Rank-Sum Test was used to determine differences across genotypes (RdV, YW, and HW4). Only metabolites with a p-value ≤ 0.01 were used for computation of subsequent data analysis. The secondary metabolites were quantified according to the internal standard method^95^, and the values are expressed as per mil equivalent of the dry weight (‰ equ. dw). Hierarchical clustering and PCA analysis were generated by the same R packages used for the sequencing data^91^.

### Real-time RT-PCR analysis

The same total, DNAse I treated RNA used for RNA-sequencing, was also used to confirm candidate gene expression by RT-PCR analysis. First strand cDNA synthesis was performed in 20 μl reactions, containing 1 μg of total RNA template, 10 μM of Oligo (dT) 15 primers, and 200 U M-MLV reverse transcriptase (both Promega, Mannheim, Germany) according to the manufacturer’s instructions. Analyses were carried out on a StepOnePlus^TM^ System (Applied Biosystems^TM^, ThermoFisher Scientific, Waltham, US-MA) equipped with StepOne^TM^ software v2.1. For each PCR reaction (10 μl in total), 2 μl cDNA, 400 nM primers, and 5 μl 2x master mix (SensiFast^TM^ SYBR Hi-Rox Kit, Bioline,) were used. Prior to gene expression analysis, PCR reactions were optimized in cDNA concentration, primer concentration, and annealing temperature (*FvGT1* gene12591: 1x cDNA, 61 °C; *FvMYB10* gene31413: 0.1x cDNA, 57 °C; *FvUBC9* gene12591: 0.1x cDNA, 55 °C; 400 nM primers for all three genes). The efficiency of each primer pair was determined using the standard curve of a serial cDNA dilution. Several possible reference genes from literature were tested, but in the end only *FvUBC9* was suitable, amplified by a primer set according to literature^54^ (Table S6). It was, however, differentially expressed between achenes and receptacle, but showed uniform levels within the respective tissue (Figure S3). Achenes and receptacle samples were, consequently, normalized separately. The cycling program was 2 min at 95 °C, followed by 40 cycles of 5 sec at 95 °C, 10 sec at 55–61 °C, and 20 sec at 72 °C, and ending in a melting curve detection of 15 sec at 95 °C, 1 min at 60 °C, and 15 sec at 95 °C. Analyses were performed in triplicates. Relative quantification was performed according to^99^ using UBC9 as reference gene.

## Additional Information

**How to cite this article:** Härtl, K. *et al*. Early metabolic and transcriptional variations in fruit of natural white-fruited *Fragaria vesca* genotypes. *Sci. Rep.* **7**, 45113; doi: 10.1038/srep45113 (2017).

**Publisher's note:** Springer Nature remains neutral with regard to jurisdictional claims in published maps and institutional affiliations.

## Supplementary Material

Supplementary Dataset

Supplementary Tables and Figures

## Figures and Tables

**Table 1:** Fold change (logFC) of genes significantly down-regulated in both white genotypes *F. vesca* YW and HW4 compared to the red genotype RdV.

Gene_ID	logFC	SUM RdV	SUM YW	SUM HW4	Prediction	Confirmed function
gene00395	−1.9	732	315	151	protein ZINC INDUCED FACILITATOR-LIKE 1-like (LOC101299619)	
gene01839	−1.9	10,330	3,811	2,475	probable cinnamyl alcohol dehydrogenase 1 (LOC101292655)	
gene04905	−3.7	98	50	2	receptor-like protein 12 (LOC101309371)	
gene05464	−7.8	45	2	0	uncharacterized sequence	
gene06602	−1.7	2,566	922	710	crocetin glucosyltransferase, chloroplastic-like (LOC101309923)	
gene07846	−4.2	35	47	0	pentatricopeptide repeat-containing protein At1g12300, mitochondrial-like (LOC101315323)	
gene08163	−5.0	58	30	0	uncharacterized sequence	
gene10142	−5.0	1,112	113	157	2-alkenal reductase NADP(+)-dependent-like (LOC101302097)	
gene12477	−6.1	20	0	0	12-oxophytodienoate reductase 3-like (LOC101293338)	
gene12565	−4.2	1,996	591	187	S-norcoclaurine synthase-like (LOC101292845)	
**gene12591**	−9.2	2,341	10	6	anthocyanidin 3-O-glucosyltransferase 2 (LOC101300000)	**GT1^27^**
gene12759	−3.4	310	370	3	putative F-box/LRR-repeat protein 23 (LOC101304436)	
gene12884	−4.0	394	4	95	dirigent protein 1-like (LOC101292468)	
gene13009	−4.3	76	12	3	F-box protein CPR30-like (LOC101302499)	
**gene14611**	−2.4	2,517	665	763	naringenin, 2-oxoglutarate 3-dioxygenase (LOC101300182)	**FHT^6^**
**gene15176**	−3.6	715	307	133	bifunctional dihydroflavonol 4-reductase/flavanone 4-reductase (DFR) (LOC101293459)	**DFR^37^**
gene16103	−1.4	771	318	268	pyridoxal kinase (LOC101304704)	
gene16795	−4.7	58	53	0	uncharacterized sequence	
gene17181	−5.7	8	0	0	lysine histidine transporter-like 8 (LOC101292649)	
gene20261	−8.0	45	3	0	TMV resistance protein N-like (LOC101312392)	
**gene21346**	−2.2	1,209	729	319	probable chalcone--flavonone isomerase 3 (LOC101305307)	**CHI^10^**
gene23269	−2.7	221	277	4	uncharacterized sequence	
gene24010	−4.6	54	7	10	uncharacterized sequence	
gene24179	−1.7	648	202	160	aspartic proteinase Asp1 (LOC101314219)	
gene25083	−4.9	33	1	8	12-oxophytodienoate reductase 2-like (LOC101297812)	
**gene26826**	−3.2	1,309	407	246	polyketide synthase 1 (LOC101298456)	**FvCHS2-2^35^**
gene27955	−4.9	69	6	1	receptor-like serine/threonine-protein kinase SD1-8 (LOC101306554)	
gene30678	−4.1	188	179	1	transmembrane protein 184 homolog DDB_G0279555-like (LOC105350765)	
gene31672	−7.2	678	17	1	glutathione S-transferase F11-like (LOC101294111)	
**gene32347**	−3.3	1,161	253	205	leucoanthocyanidin dioxygenase (LOC101308284)	**ANS^6^**
gene32421	−3.0	642	92	88	protein P21-like (LOC101300343)	
gene32435	−2.5	551	198	92	short-chain dehydrogenase/reductase 2b-like (LOC101296098)	
gene33838	−1.4	291	188	57	AMP deaminase-like (LOC101301583)	

**Table 2:** Fold change (logFC) of genes significantly up-regulated in both white genotypes *F. vesca* YW and HW4 compared to the red genotype RdV.

Gene_ID	logFC	SUM RdV	SUM YW	SUM HW4	Prediction
gene01275	6.7	0	7	8	uncharacterized sequence
gene03760	5.3	4	67	87	ceramide−1-phosphate transfer protein (LOC101298698)
gene04372	4.4	1	1	150	mitochondrial saccharopine dehydrogenase-like oxidoreductase At5g39410 (LOC101312472)
gene07901	2.4	126	633	441	18.1 kDa class I heat shock protein-like (LOC101300322)
gene08062	7.1	0	10	10	CDT1-like protein b (LOC101298288)
gene08537	2.5	82	339	403	uncharacterized LOC101304935 (LOC101304935)
gene09254	4.5	3	8	442	uncharacterized LOC101298543 (LOC101298543)
gene12602	6.4	0	1	99	uncharacterized LOC101291726 (LOC101291726)
gene12786	3.8	4	8	138	B3 domain-containing transcription factor VRN1-like (LOC101297387)
gene13191	1.5	1,127	2,885	3,654	heat shock protein 83 (LOC101307345)
gene13320	2.1	63	207	337	BCL2-associated athanogene 3 (BAG3)
gene16235	2.5	69	232	472	homeobox-leucine zipper protein ATHB-6-like (LOC101309384)
gene16479	5.0	0	16	19	cysteine synthase-like (LOC101302477)
gene16510	4.4	2	27	41	uncharacterized LOC101294957 (LOC101294957)
gene19533	7.2	0	4	35	putative receptor-like protein kinase At4g00960 (LOC105350176)
gene20844	9.1	0	15	99	uncharacterized LOC105353058 (LOC105353058)
gene20847	10.7	6	1,038	5,630	calmodulin-interacting protein 111-like (LOC101311429)
gene24034	5.2	5	68	142	uncharacterized LOC101294593 (LOC101294593)
gene24512	11.3	0	159	225	uncharacterized LOC101301427 (LOC101301427)
gene24545	2.8	56	335	226	uncharacterized LOC101302298 (LOC101302298)
gene24775	5.8	0	3	10	uncharacterized LOC101301427 (LOC101301427)
gene24779	7.1	0	8	18	uncharacterized LOC101302918 (LOC101302918)
gene26609	2.9	16	74	140	dolichyl-phosphate beta-glucosyltransferase (LOC101312675)
gene27422	10.3	0	25	426	transcription factor ORG2-like (LOC101309207)
gene27944	6.0	0	0	67	uncharacterized LOC101309177 (LOC101309177)
gene27945	4.9	0	0	209	uncharacterized LOC101305101 (LOC101305101)
gene28620	8.7	0	20	43	trifunctional UDP-glucose 4,6-dehydratase/UDP-4-keto-6-deoxy-D-glucose 3,5-epimerase/UDP-4-keto-L-rhamnose-reductase RHM1-like (LOC101302674)
gene29781	7.2	0	15	11	anthocyanidin 3-O-glucoside 2″-O-glucosyltransferase-like (LOC101310006)
gene30676	7.2	0	0	522	uncharacterized sequence
gene30960	4.6	110	743	4,114	uncharacterized sequence
gene32014	5.9	0	6	3	ABC transporter C family member 10-like (LOC101302270)

**Table 3:** Flavonoid genes differentially expressed in the red- and white-fruited genotypes and transcription factors analyzed in this study.

GT1	gene12591	XM_004307828
F3H	gene14611	XM_004287766
DFR	gene15176	XM_004291810
CHI	gene21346	XM_004307686
CHS2-2	gene26826	XM_004306495
ANS	gene32347	XM_004298672
GST	gene31672	XM_004288530
ORG2 (bHLH)	gene27422	XM_004290363
MYB10	gene31413	XM_004302169
MYB1	gene09407	XM_004299494
