# Supplementary material for: Early metabolic and transcriptional variations in fruit of natural white-fruited Fragaria vesca genotypes
Source: Sci Rep. 2017 Mar 22;7:45113. doi: 10.1038/srep45113 (PMC5361166; doi:10.1038/srep45113)
Supplement: Supplementary Tables and Figures [file srep45113-s2.pdf]

Supplements

Early metabolic and transcriptional variations in fruit of natural white-fruited *Fragaria vesca* genotypes

Katja Härtl[1], Alisandra Denton[2], Katrin Franz-Oberdorf[1], Thomas Hoffmann[1], Melanie Spornraft[3], Björn Usadel[2], Wilfried Schwab[1]*

[1] Biotechnology of Natural Products, Technische Univeristät München, Liesel-Beckmann-Str. 1, 85354 Freising, Germany

[2] Institute for Biology I, RWTH Aachen University, Worringer Weg 3, 52074 Aachen, Germany

[3] Physiology Weihenstephan, TU München, Weihenstephaner Berg 3, 85354 Freising, Germany

Tables

Table S1: RIN – values

ID	Strawberry Variety	Ripening Stage	Tissue	RIN
1	"Reine des Vallees"	ripe	Receptacle	8.5
2			Achenes	6.2
3		intermediate	Receptacle	8.5
4			Achenes	7.0
5		green	Receptacle	8.7
6			Achenes	7.1
7	"Yellow Wonder"	ripe	Receptacle	8.7
8			Achenes	8.3
9		intermediate	Receptacle	8.3
10			Achenes	7.2
11		green	Receptacle	7.0
12			Achenes	8.3
13	"Hawaii 4"	ripe	Receptacle	9.3
14			Achenes	7.9
15		intermediate	Receptacle	8.6
16			Achenes	7.5
17		green	Receptacle	8.3
18			Achenes	7.7

Table S2: Sequencing Results; Sequences are demultiplexed according to the 6 bp index code with 0 mismatch allowed. "Yield (Mbp)" specifies the number of bases called in mega bases. All reads have passed the Illumina chastity filter. "%Q30" represents the percentage of bases with a quality score of at least 30 (inferred base call accuracy of 99.9%). The Q-score is a prediction of the probability of a wrong base call.

Lane	ID	Index	Yield (Mbp)	#Cluster	%Q30	Mean Q
2	1	ATCACG	1,921	19,205,021	95.49	36.97
2	2	CGATGT	1,181	11,811,431	96.66	37.32
2	3	TTAGGC	1,264	12,635,961	96.18	37.20
2	4	TGACCA	1,454	14,539,863	95.61	36.97
2	5	ACAGTG	2,058	20,584,618	96.66	37.38
2	6	GCCAAT	1,156	11,556,879	96.58	37.34
2	7	CAGATC	1,350	13,500,409	96.56	37.31
2	8	ACTTGA	1,932	19,322,039	95.86	37.04
2	9	GATCAG	1,568	15,681,771	96.39	37.28
2			∑ 13,884	∑ 138,837,992		
3	10	ATCACG	1,200	11,997,650	97.23	37.53
3	11	CGATGT	1,086	10,863,231	97.41	37.59
3	12	TTAGGC	1,180	11,798,844	97.24	37.53
3	13	TGACCA	1,307	13,072,075	97.43	37.67
3	14	ACAGTG	1,217	12,167,220	97.14	37.49
3	15	GCCAAT	1,237	12,366,260	97.02	37.49
3	16	CAGATC	1,255	12,545,408	97.13	37.45
3	17	ACTTGA	1,321	13,210,822	95.82	36.98
3	18	GATCAG	1,272	12,722,858	97.35	37.58
3			∑ 11,075	∑ 110,744,368		
			**∑ 24,959**	**∑ 249,582,360**		

Table S3: Read Mapping Results

ID	Input	Mapped	Rate [%]
1	19,127,187	14,995,534	78.4
2	11,765,186	10,191,209	86.6
3	12,584,685	10,251,152	81.5
4	14,481,463	10,963,855	75.7
5	20,505,771	17,115,834	83.5
6	11,512,157	9,757,056	84.8
7	13,450,559	10,700,255	79.6
8	19,244,492	15,093,497	78.4
9	15,620,144	12,971,680	83.0
10	11,964,382	10,300,023	86.1
11	10,831,642	9,786,653	90.4
12	11,763,556	10,621,766	90.3
13	13,032,881	11,963,684	91.8
14	12,131,564	10,711,818	88.3
15	12,328,783	10,643,386	86.3
16	9,402,323	8,214,526	87.4
17	13,170,437	8,933,261	67.8
18	12,686,615	11,673,334	92.0
	∑ 245,603,827	∑ 204,888,523	∑ 83.4

Table S4: Levels of secondary metabolites in per mil equivalents of the dry weight (‰ equ. dw.) measured in **achene** tissues of strawberry fruits in green, intermediate and ripe developmental stage.

Metabolite	**RdV green Ac**	**RdV inter Ac**	**RdV ripe Ac**	**YW green Ac**	**YW inter Ac**	**YW ripe Ac**	**HW4 green Ac**	**HW4 inter Ac**	**HW4 ripe Ac**
Gallic acid	0.08±0.01	0.04±0.01	0.04±0.02	0.07±0.03	0.02±0.02	0.02±0.01	0.06±0.02	0.05±0.01	0.04±0.01
Gallic acid glucose ester	0.17±0.03	0.14±0.03	0.16±0.08	0.19±0.08	0.06±0.04	0.09±0.02	0.21±0.06	0.16±0.03	0.12±0.03
Ellagic acid	1.80±0.69	1.28±0.30	1.38±0.93	1.83±0.93	1.56±0.44	1.77±0.61	1.34±0.59	1.92±0.35	1.70±0.44
Cinnamic acid glucose ester	0.00±0.00	0.00±0.00	0.00±0.00	0.00±0.00	0.00±0.00	0.00±0.00	0.00±0.00	0.00±0.00	0.00±0.00
4-Coumaric acid glucose ester	0.00±0.00	0.00±0.00	0.07±0.02	0.00±0.00	0.00±0.00	0.00±0.00	0.00±0.00	0.00±0.00	0.00±0.00
Caffeic acid glucose ester	0.00±0.00	0.00±0.00	0.05±0.01	0.00±0.00	0.02±0.00	0.04±0.00	0.00±0.00	0.00±0.00	0.03±0.01
Quercetin glucuronide	0.00±0.00	0.00±0.00	0.00±0.00	0.00±0.00	0.00±0.00	0.00±0.00	0.00±0.00	0.00±0.00	0.00±0.00
Kaempferol glucuronide	1.37±0.15	1.73±0.34	1.85±0.28	1.17±0.33	1.77±0.53	1.82±0.31	1.05±0.25	1.62±0.21	1.66±0.38
Kaempferol glucoside	0.24±0.01	0.38±0.12	0.37±0.09	0.12±0.05	0.11±0.12	0.20±0.16	0.14±0.05	0.22±0.03	0.21±0.08
Catechin	0.25±0.08	0.37±0.15	0.28±0.15	0.14±0.05	0.14±0.07	0.30±0.06	0.25±0.04	0.19±0.07	0.18±0.04
Epicatechin catechin dimers	0.70±0.01	0.88±0.22	0.71±0.22	0.50±0.12	0.44±0.06	0.99±0.19	0.55±0.13	0.46±0.11	0.52±0.17
Epiafzelechin catechin dimers	0.42±0.04	0.51±0.15	0.48±0.16	0.27±0.09	0.25±0.04	0.59±0.14	0.37±0.14	0.34±0.07	0.40±0.16
Pelargonidin glucoside	0.00±0.00	0.21±0.06	4.50±1.78	0.00±0.00	0.00±0.00	0.00±0.00	0.00±0.00	0.00±0.00	0.00±0.00
Pelargonidin glucoside malonate	0.00±0.00	0.33±0.11	3.85±1.57	0.00±0.00	0.00±0.00	0.00±0.00	0.00±0.00	0.00±0.00	0.00±0.00
Cyanidin glucoside	0.00±0.00	0.38±0.10	3.26±1.35	0.00±0.00	0.00±0.00	0.00±0.00	0.00±0.00	0.00±0.00	0.00±0.00

Table S5: Levels of secondary metabolites in per mil equivalents of the dry weight (‰ equ. dw.) measured **in receptacle** tissues of strawberry fruits in green, intermediate and ripe developmental stage.

Metabolite	**RdV green Rc**	**RdV inter Rc**	**RdV ripe Rc**	**YW green Rc**	**YW inter Rc**	**YW ripe Rc**	**HW4 green Rc**	**HW4 inter Rc**	**HW4 ripe Rc**
Gallic acid	0.00±0.00	0.00±0.00	0.00±0.00	0.00±0.00	0.00±0.00	0.00±0.00	0.00±0.00	0.00±0.00	0.00±0.00
Gallic acid glucose ester	0.00±0.00	0.00±0.00	0.00±0.00	0.00±0.00	0.00±0.00	0.00±0.00	0.00±0.00	0.00±0.00	0.00±0.00
Ellagic acid	0.13±0.02	0.09±0.02	0.28±0.05	0.30±0.03	0.18±0.02	0.24±0.07	0.27±0.04	0.17±0.03	0.24±0.05
Cinnamic acid glucose ester	0.00±0.00	0.01±0.00	0.15±0.02	0.00±0.00	0.00±0.00	0.04±0.00	0.00±0.00	0.00±0.00	0.05±0.01
4-Coumaric acid glucose ester	0.00±0.00	0.04±0.01	0.59±0.13	0.00±0.00	0.00±0.00	0.00±0.00	0.00±0.00	0.00±0.00	0.05±0.01
Caffeic acid glucose ester	0.00±0.00	0.00±0.00	0.00±0.00	0.00±0.00	0.00±0.00	0.00±0.00	0.00±0.00	0.00±0.00	0.00±0.00
Quercetin glucuronide	0.15±0.03	0.10±0.02	0.25±0.02	0.24±0.03	0.15±0.01	0.10±0.01	0.18±0.04	0.13±0.03	0.11±0.02
Kaempferol glucuronide	0.48±0.11	0.45±0.03	0.59±0.15	1.12±0.15	0.69±0.02	0.63±0.09	0.80±0.13	0.55±0.05	0.58±0.05
Kaempferol glucoside	0.03±0.01	0.03±0.00	0.08±0.02	0.09±0.04	0.16±0.20	0.22±0.16	0.05±0.01	0.04±0.01	0.04±0.01
Catechin	1.83±0.24	1.71±0.33	1.59±0.31	2.75±0.45	1.72±0.09	1.49±0.27	2.78±0.44	1.92±0.11	1.58±0.20
Epicatechin catechin dimers	4.60±0.42	6.30±0.93	5.13±0.67	8.03±1.05	5.75±0.17	4.88±0.83	6.11±0.60	6.71±0.53	4.77±0.30
Epiafzelechin catechin dimers	0.64±0.08	1.00±0.14	0.68±0.10	1.09±0.23	0.81±0.03	0.34±0.08	0.73±0.05	0.96±0.09	0.55±0.08
Pelargonidin glucoside	0.00±0.00	0.04±0.01	4.68±0.51	0.00±0.00	0.00±0.00	0.00±0.00	0.00±0.00	0.00±0.00	0.00±0.00
Pelargonidin glucoside malonate	0.00±0.00	0.00±0.00	0.14±0.03	0.00±0.00	0.00±0.00	0.00±0.00	0.00±0.00	0.00±0.00	0.00±0.00
Cyanidin glucoside	0.00±0.00	0.08±0.03	3.16±0.35	0.00±0.00	0.00±0.00	0.00±0.00	0.00±0.00	0.00±0.00	0.00±0.00

Table S6: RT-PCR primers

Gene	Nomenclature	Sequence	Reference
FvGT1	fw	CACTCACGCCGCCCCACTG	[27]
	rev	CACACCATCCGCCACCTCACAC	
FvMYB10	fw	TCAAATCAGGCTTAAACAGA	[54]
	rev	TTAAAGACCACCTGTTTCCT	
FvUBC9	fw	ATCTGCTCACTGTTGACGGA	[54]
	rev	AGCTCCTTGCTGTTGTCTCA	

Figures

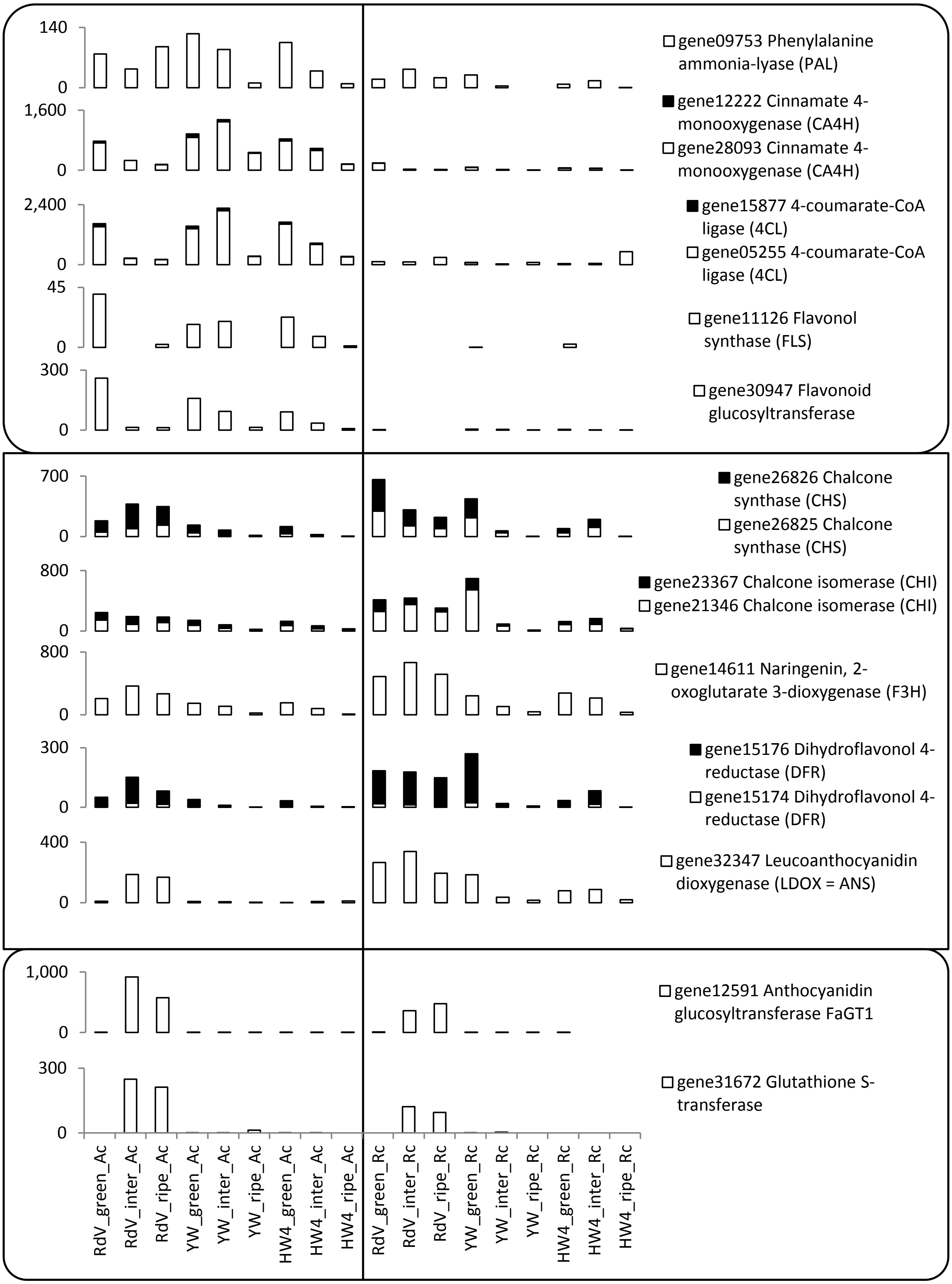

Figure S1: Transcript levels (normalized RPM) of genes encoding enzymes involved in flavonoid/anthocyanin formation in receptacle (Rc) and achenes (Ac) of *F. vesca* cv. RdV, YW, and HW4 at the green, intermediate and ripe developmental stage.

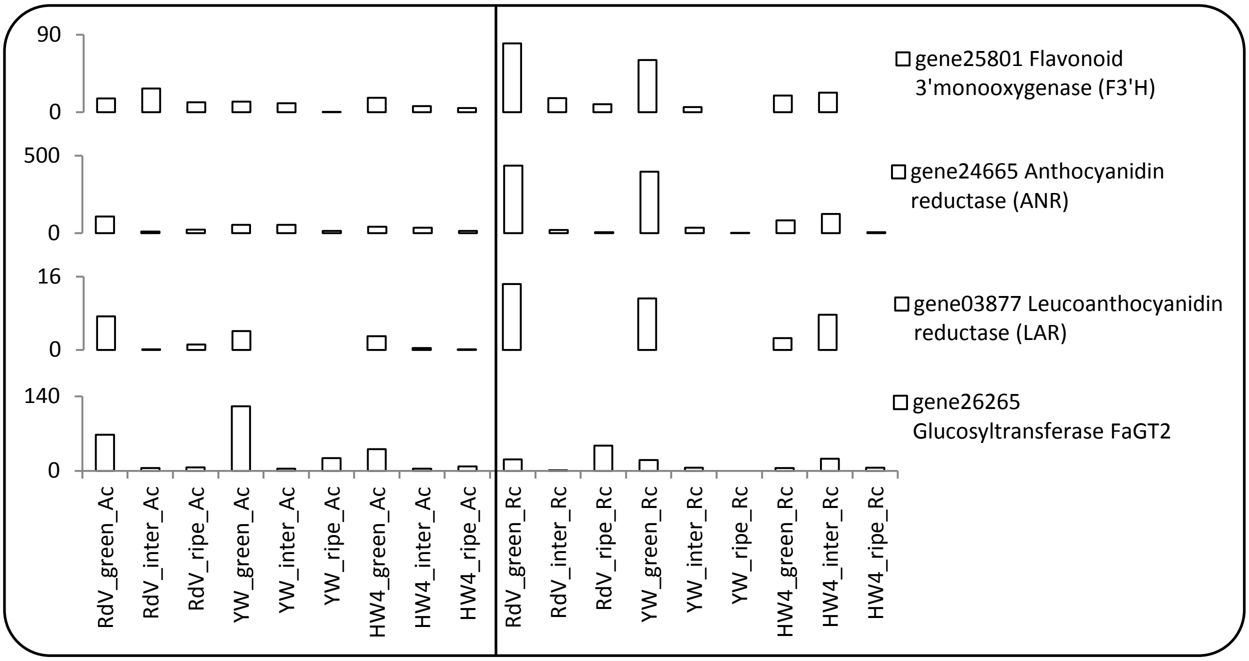

Figure S2: Transcript levels (normalized RPM) of genes encoding enzymes involved in proanthocyanidin, and *β*-glucogallin formation in receptacle (Rc) and achenes (Ac) of *F. vesca* cv. RdV, YW, and HW4 at the green, intermediate and ripe developmental stage.

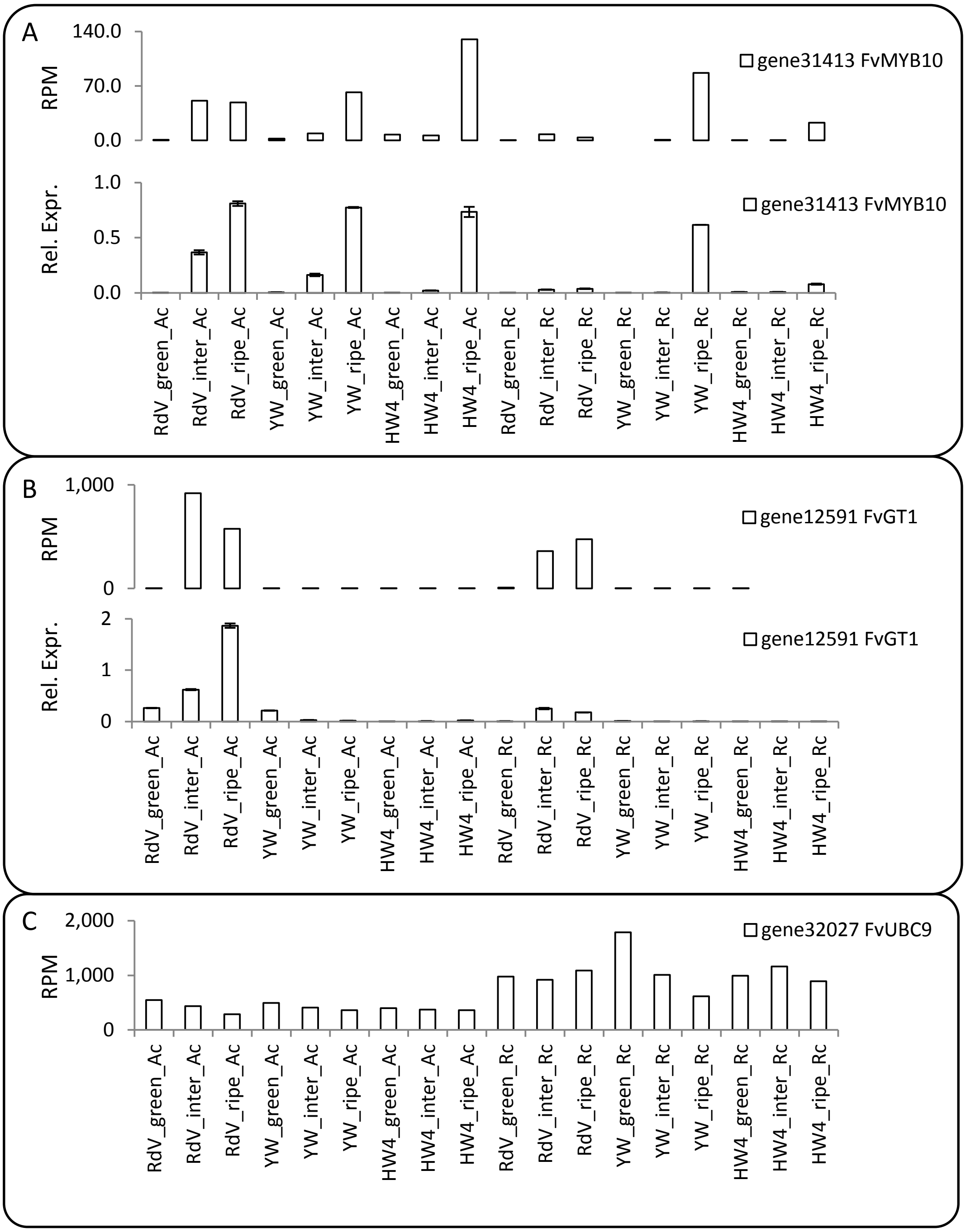

Figure S3: Comparison of gene transcript levels determined by RNA sequencing (RPM) and RT-PCR (relative Expression, Rel. Expr.) for *MYB10* (A) and *GT1* (B), and RPM of the reference gene *UBC9* (C) in receptacle (Rc) and achenes (Ac) of *F. vesca* RdV, YW, and HW4 at the green, intermediate, and ripe developmental stage.
